# *Campylobacter jejuni* 11168H Exposed to Penicillin Forms Persister Cells and Cells With Altered Redox Protein Activity

**DOI:** 10.3389/fcimb.2020.565975

**Published:** 2020-10-19

**Authors:** Helen Morcrette, Andrea Kovacs-Simon, Richard K. Tennant, John Love, Sariqa Wagley, Zheng R. Yang, David J. Studholme, Orkun S. Soyer, Olivia L. Champion, Clive S. Butler, Richard W. Titball

**Affiliations:** ^1^College of Life and Environmental Sciences—Biosciences, University of Exeter, Exeter, United Kingdom; ^2^School of Life Sciences, University of Warwick, Coventry, United Kingdom

**Keywords:** *Campylobacter jejuni*, proteomics, persister cell, antibiotic, electron transport

## Abstract

The formation of persister cells is one mechanism by which bacteria can survive exposure to environmental stresses. We show that *Campylobacter jejuni* 11168H forms persister cells at a frequency of 10^−3^ after exposure to 100 × MIC of penicillin G for 24 h. Staining the cell population with a redox sensitive fluorescent dye revealed that penicillin G treatment resulted in the appearance of a population of cells with increased fluorescence. We present evidence, to show this could be a consequence of increased redox protein activity in, or associated with, the electron transport chain. These data suggest that a population of penicillin G treated *C. jejuni* cells could undergo a remodeling of the electron transport chain in order to moderate membrane hyperpolarization and intracellular alkalization; thus reducing the antibiotic efficacy and potentially assisting in persister cell formation.

## Introduction

*Campylobacter jejuni* is the leading bacterial cause of gastroenteritis in the world estimated be causing almost 100 million cases worldwide (Asuming-Bediako et al., 2019) with 1 million cases a year in the US, and over 250,000 cases in the European Union (Chlebicz and Slizewska, 2018). In many areas of the world the reported incidence of disease appears to be increasing (Chlebicz and Slizewska, 2018). The avian gut is the normal site of carriage of the bacterium and most cases in humans arise from the ingestion of contaminated poultry (Chlebicz and Slizewska, 2018; Igwaran and Okoh, 2019; Rossler et al., 2019). In developing countries, *Campylobacter* is hyper-endemic, a leading bacterial cause of diarrhoeal disease and a major cause of infant mortality (Asuming-Bediako et al., 2019; Igwaran and Okoh, 2019). The bacterium is notoriously fastidious and survives poorly under laboratory conditions, yet it appears to be ubiquitous in the environment, forming reservoirs of infection (Murphy et al., 2006; Bronowski et al., 2014). Environmental survival of the bacterium is essential for transmission to new hosts. Survival outside of the host is also evidenced in the ability of the bacterium to tolerate abiotic stresses encountered during food processing (Ligowska et al., 2011; Garcia-Sanchez et al., 2017).

One mechanism employed by bacteria to survive otherwise lethal stresses is the formation of persister cells (Lewis, 2005, 2007). Persistence has been defined as the ability of a subset of the population (perisister cells) to survive exposure to a bactericidal drug concentration (Balaban et al., 2019). It is an example of phenotypic switching; the ability of a genetically identical population of organisms to display diverse phenotypes under a given environment (Balaban et al., 2004; Lewis, 2007). There is good evidence that persister cells are present in the bacterial population before exposure to the stress. They can be revealed by exposing the population to a supra-lethal dose of a bactericidal antibiotic (Lewis, 2007; Balaban et al., 2019), which kills most of the bacterial population, except for the persister cells. The ability of persister cells to survive exposure to abiotic stresses, including multiples of the minimum inhibitory concentration (MIC) of antibiotics, biocides and killing by toxic metals, is ascribed to their low growth rates or dormancy (Harrison et al., 2005; Lewis, 2008; Harms et al., 2016). There is also increasing evidence that persister cells make up a subpopulation of antibiotic-resistant cells in biofilms (Lewis, 2007, 2008; Jayaraman, 2008; Harrison et al., 2009; Kim et al., 2009).

The mechanisms by which persister cells form and their molecular makeup have been studied intensively over the past few years and reveal some common mechanisms but have also revealed that a diverse range of molecular events are associated with persister cell formation (Wilmaerts et al., 2019). Toxin-antitoxin systems may play a role by regulating metabolism and targeting functions such as transcription, translation and DNA replication (Harms et al., 2016). Another mechanism involves energy metabolism: a number of reports linking the electron transport chain to persister cell formation (Harms et al., 2016). Drug efflux pumps may contribute to their formation too (Harms et al., 2016). We have previously shown that oxygen availability can influence persister cell formation (Hemsley et al., 2014). At the time of submitting this manuscript there were no reports of the formation of persister cells by *Campylobacter*.

Here, we set out to investigate whether *C. jejuni* 11168H is able to form persister cells and to investigate the molecular makeup of these cells. *C. jejuni* strain 11168H is a strain, selected as representative, for the first Campylobacter genome sequencing project (Parkhill et al., 2000). This should provide new insight into the mechanisms by which *C. jejuni* 11168H survives exposure to antibiotic stresses and could reveal mechanisms that it uses to survive in the environment.

## Materials and Methods

### Growth of *C. jejuni*

*C. jejuni* 11168H was cultured on Columbia agar plates (CBA) supplemented with either 5–9% (v/v) horse blood or with Skirrow selective supplement (Oxoid Ltd., Basingstoke, UK) and 5–9% (v/v) horse blood in a variable atmosphere incubator (VAIN) (Don Whitley Scientific, Bingley, UK) under microaerobic conditions (5% O_2_, 85% N_2_, 10% CO_2_) at 37°C for 24 or 48 h. The bacteria were sub-cultured into 25 ml of Mueller-Hinton broth (Oxoid) and grown under microaerobic conditions as before (Champion et al., 2010).

### Staining With BacLight™

Bacteria were enumerated using *Bac*Light™ Live/Dead Bacterial Viability Kit (Life Technologies, Paisley, UK) according to the manufacturers' instructions. Previous studies have demonstrated the utility of *Bac*Light™ for the differentiation of live and dead *C. jejuni* (Alonso et al., 2002; He and Chen, 2010; Kim et al., 2014). Briefly, aliquots of bacterial cells were treated with premixed stain, mixed and incubated for 15 min in the dark. Two μl of the resulting bacterial suspension was then placed on poly-L-lysine coated glass slides (Sigma, Dorset, UK) along with 2 μl of 1 μm polystyrene latex beads (Sigma), and fluorescence viewed using a Zeiss Fluorescence Microscope with an FITC filter. The total number of cells and the number of specifically stained cells was calculated using Image J software (Schneider et al., 2012).

### Staining With Redox Sensor Green

Aliquots of the bacterial cell suspension in 1 ml PBS were stained with 4 μl of *Bac*Light™ Redox Sensor Green reagent (RSG) (Life Technologies) for 10 min in the dark at room temperature and then visualized after excitation at 490 nm by microscopy as described above or flow cytometry as detailed below.

### Exposure to Penicillin G

The MIC of penicillin G toward *C. jejuni* 11168H was measured using ETEST® antimicrobial susceptibility test strips (Biomerieux, Basingstoke, UK). Overnight broth cultures were diluted to the equivalent of a McFarland turbidity standard of 0.5 and used to create a lawn on Mueller-Hinton agar plates. The ETEST^®^ strips were added and the plates were incubated at 37°C for 24 h under microaerobic conditions and zones of growth inhibition recorded. We found that the MIC of penicillin G was 11.43 μg/ml. For studies where *C. jejuni* 11168H was exposed to penicillin G, bacteria were first grown in MHB broth for 14 h and the cell harvested by centrifugation and re-suspended in fresh MHB or in MHB containing 100 × the MIC of penicillin G (1,143 μg/ml).

### Bacterial Killing by Penicillin G

Broth cultured bacteria were incubated for 24 h with either 100 × the MIC of penicillin G (Sigma) in Mueller Hinton broth or in Mueller Hinton broth alone. At intervals, samples of the cultures were taken, centrifuged and the cell pellet re-suspended in phosphate buffered saline (PBS; pH 7.2). Bacteria were then enumerated as described above. To calculate the number of colony forming units (CFU), bacteria were serially diluted in PBS, plated onto Mueller Hinton agar plates (Oxoid) and incubated at 37°C under microaerobic conditions. The frequency of persister cell formation was calculated as the number of *C. jejuni* cells cultured after 24 h exposure to 100 × MIC of penicillin/number of *C. jejuni* cells cultured before exposure to antibiotic. Assays were carried out in triplicate.

### Flow Cytometry

Aliquots of untreated and penicillin G-treated *C. jejuni* 11168H were stained with Redox Sensor Green as described above, and analyzed in a BD FACS Aria III (Becton Dickenson fluorescence-activated cell sorting (FACS) cytometer using a 488 nm laser. Emission of fluorescence was detected at 530 ± 30 nm. Three distinct RSG stained populations (bright, dim and unstained, 3 × 10^6^ cells) were sorted into 10 ml falcon tubes and kept on ice.

The populations of bacterial cells were collected as described above, concentrated by centrifugation at 11,337 × g for 30 min at 4°C and then pooled and lysed in 70 μl of Bugbuster reagent (Merck Millipore, Dorset, UK). After shaking at room temperature for 20 min the bacterial lysates were stored at −80°C.

### Mass Spectrometry

Proteomics was performed as described previously (Goggs et al., 2013). Briefly, an UltiMate™3000 nano HPLC system in line with an LTQ-Orbitrap Velos mass spectrometer (Thermo Scientific) was used. The raw data files were processed and quantified using Proteome Discoverer software v1.2 (Thermo Scientific) and searched against UniProt *C. jejuni* strain 11168H database using the SEQUEST algorithm. The reverse database search option was enabled and all peptide data was filtered to satisfy false discovery rate (FDR) of 5%. Abundance of each protein in each sample was calculated using the average area measurements of the three most abundant peptides matching to each protein (Top3 method) (Ahrne et al., 2013). Normalization of the mass spectrometric data (protein abundances) was performed globally at protein level. Abundance of each protein was expressed as the fraction of the signal derived from the total abundance detected in each sample (Ting et al., 2009). This value was then compared for each protein in the penicillin G treated and control samples.

Proteins with significantly differential abundance were identified using the R packages limma and *q*-value (Smyth, 2004), when proteins with constant abundance across replicates were removed from the analysis. The former package was used for significance analysis, calculating *p*-values. The latter package was used for false discovery rate control, calculating *q*-values. Proteins with a *q* < 0.05 and more than 2-fold change difference in abundance were considered significant.

### Online Tools

Cellular localization of the proteins encoded in the *C. jejuni* strain 11168H genome was predicted using PSORTb v.3.0.2 (https://www.psort.org/psortb/) (Yu et al., 2010). *C. jejuni* strain 11168H proteins were classified into functional categories based on clusters of orthologous gene (COG) designations; COG categories were assigned to each protein using eggNOG-mapper (http://eggnog-mapper.embl.de/) (Huerta-Cepas et al., 2017, 2019).

## Results and Discussion

### *C. jejuni* 11168H Forms Persister Cells

We exposed *C. jejuni* 11168H to penicillin G, because beta-lactam antibiotics were previously used successfully to reveal persister cells in other species (Balaban et al., 2019) and globally the penicillins are widely used in poultry farming, as growth promoters and to treat disease (Allen and Stanton, 2014; Manyi-Loh et al., 2018). Therefore, *C. jejuni* in the avian gut could be exposed to high doses of penicillin.

**Figure 1:**
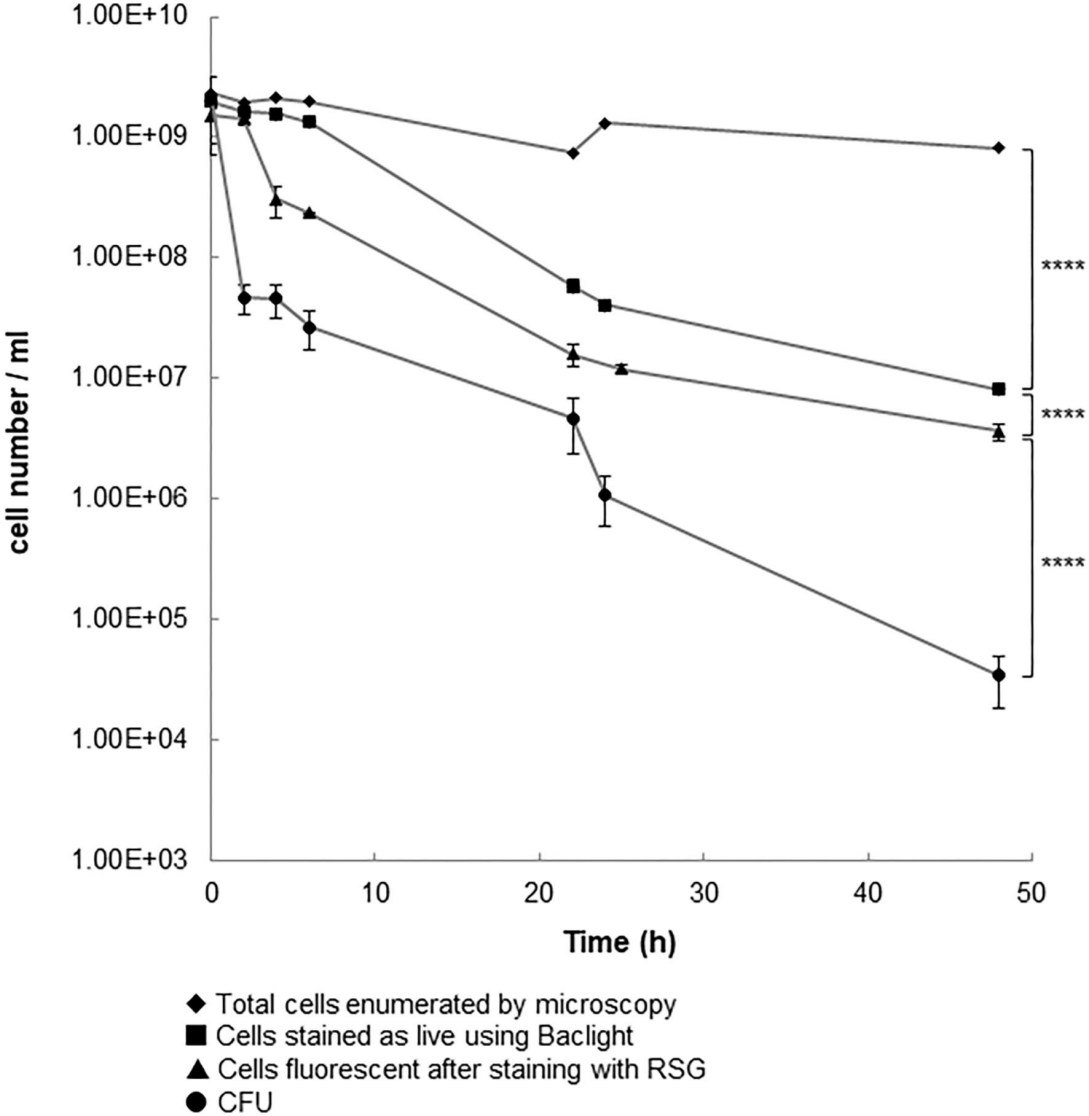
Cell populations following the addition of penicillin G to 14 h old cultures of *C. jejuni* 11168H. A log phase culture (14 h) of *C. jejuni* was incubated for 24 h with 100 × the MIC of penicillin G. Results shown are the mean of three replicates with bars corresponding to the standard error of the mean shown at each data point. ^****^p < 0.0001 (unpaired *t*-test).

We grew *C. jejuni* 11168H in broth for 14 h (mid-log phase), and then exposed cultures to 100 × the MIC (11.43 μg/ml). At intervals the numbers of total cells, culturable cells, cells strained by *Bac*Light™ and cells stained using RSG were determined for up to 48 h post dosing (Figure 1). Staining with RSG reveals metabolic activity because in metabolically active cells redox sensor green is reduced as a consequence of electron transport chain function, forming a green fluorescent dye (Jaen et al., 2019).

The numbers of culturable cells (CFU) and of cells that fluoresced after staining with RSG showed bi-phasic reductions, which is characteristic of the killing of drug-sensitive cells followed by the much slower decline in the number of drug tolerant persister cells (Lewis, 2005). The numbers of cells that stained green with *Bac*Light™ declined over the course of the experiment but did not show a biphasic reduction. When we used older cultures (24 or 48 h instead of than 14 h) for this experiment, we saw similar patterns of changes in total cells numbers, culturable cells, or *Bac*Light™ stained cells (data not shown). The total number of cells did not show the same pattern of decline. When the population that survived exposure to antibiotic was re-cultured in fresh broth, we found that the MIC of penicillin G (11 μg/ml) was similar to that of the population at the start of the experiment. This is consistent with the non-inherited and antibiotic-tolerant phenotype of persister cells. Based on the number of cells that could be cultured after exposure of the population to 100 × MIC of penicillin G for 24 h, we calculate the persister cell frequency to be 5.25 × 10^−4^. It is reported (Lewis, 2007) that the frequency of persister cell formation in other bacterial species is typically 10^−3^ to 10^−6^ (Keren et al., 2004; Lewis, 2007). The high frequency with which *C. jejuni* 11168H forms persister cells might explain the ability of this bacterium to survive a wide range of environmental insults when exposed to antibiotics. Since our manuscript was submitted Ovsepian et al. (2020) reported that *C. jejuni* strains 81–176 and RM1221 form persister cells which are revealed by exposure to ciprofloxacin at frequencies of 10^−5^ to 10^−7^ after exposure to this drug for 22 h. This finding confirms that a range of strains of *C. jejuni* can form persister cells. However, these authors were unable to demonstrate persister cells resistant to 100 × MIC of ampicillin. This could indicate strain-specific differences in persister cell formation or differences in the responses to ampicillin or penicillin G exposure. Strain-specific differences in persister cell formation and differences in survival after exposure to different drugs is well-established (Fisher et al., 2017). Further work is required to investigate these possibilities in *C. jejuni*.

### Flow Cytometry Reveals Three Populations After Exposure to Penicillin G

**Figure 2:**
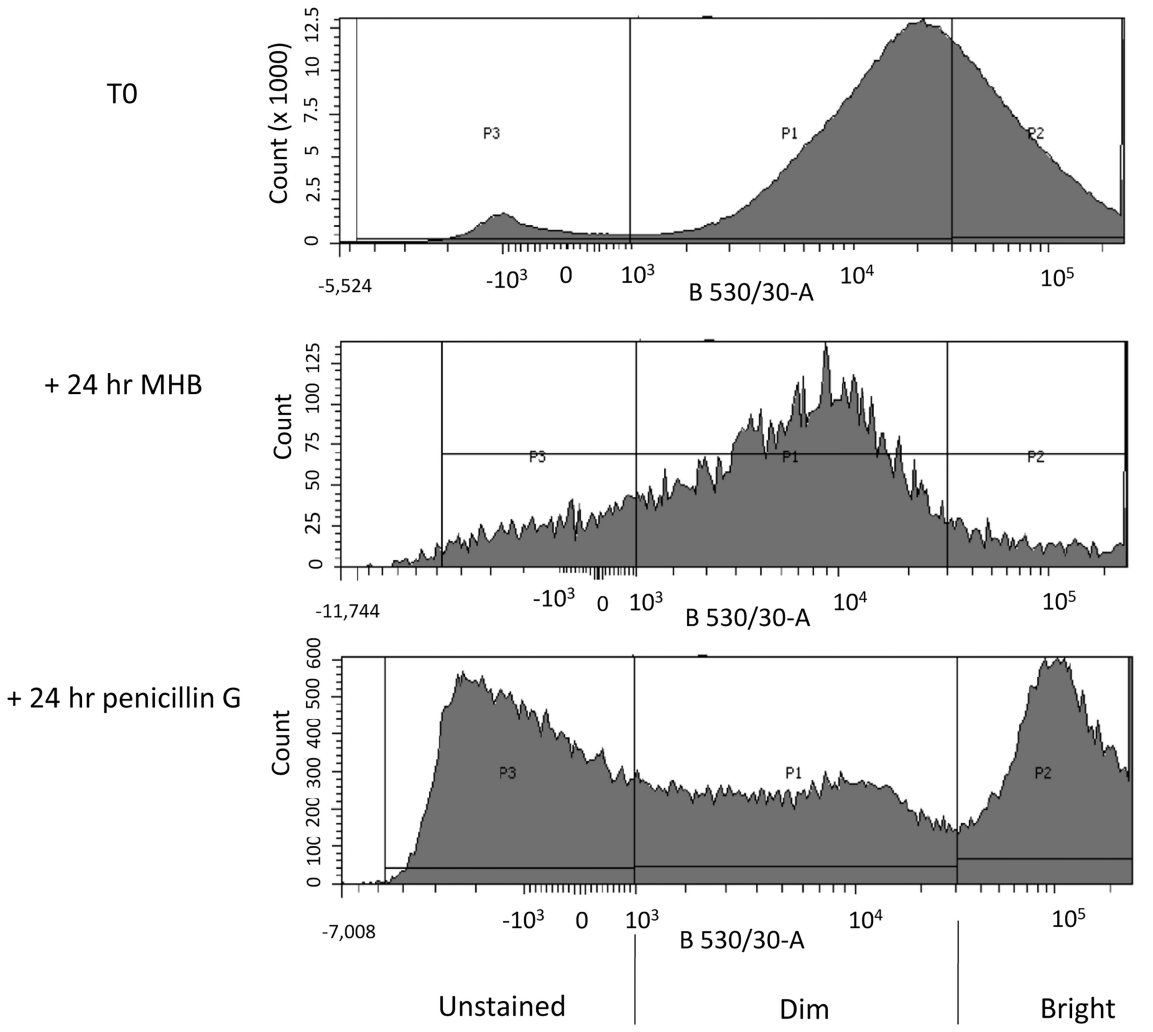
FACS profiles of *C. jejuni* 11168H after staining with RSG. Three biological replicates of each of the following: A log phase (14 h growth) *C. jejuni* culture (T0), a log phase culture incubated for a further 24 h in MHB (control + 24 h MHB) and a log phase culture incubated for a further 24 h in MHB containing 100 × MIC of penicillin G (+ 24 h Penicillin G), were stained with Redox Sensor Green (RSG). The stained cultures were then analyzed by FACS. Fluorescence intensity of RSG stained *C. jejuni* cells at 530 ± 30 nm (x-axis) after excitation with a 488 nm laser is plotted against bacterial cell count (y-axis). Different populations of cells were gated according to the intensity of the RSG fluorescence of each population (P1–stained, P2–brightly stained, and P3–unstained).

We next compared the flow cytometry histograms of cultures of *C. jejuni* 11168H that had been cultured for 14 h in MHB (T0 culture) with the histograms of cells that were subsequently incubated at 37°C under micro-aerobic conditions in either MHB or in MHB containing 100 × MIC of penicillin G. The histograms showed cells grown in MHB, had a normally distributed range of fluorescence signal intensities, ranging from dim to bright (Figure 2; T0). A small sub-population of cells were unstained.

**Table 1:** Top 20 proteins significantly over-produced (left hand side) or under-produced (right hand side) in *C. jejuni* cells that strained brightly with RSG after exposure to 100 × MIC of penicillin G for 24 h.

**Significantly over-produced proteins**				**Significantly under-produced proteins**			
**Locus**	**Gene name**	**Protein name**	**Fold change**	**Locus**	**Gene name**	**Protein name**	**Fold change**
Cj0755	*cfrA*	Ferric enterobactin uptake receptor	12.4733	Cj1430c	*rfbC*	dTDP-4-dehydrorhamnose 3,5-epimerase	∞
Cj1039	*murG*	UDP-N-acetylglucosamine–N-acetylmuramyl-(pentapeptide) pyrophosphoryl-undecaprenol N-acetylglucosamine transferase	7.8637	Cj1567c	*nuoM*	NADH-quinone oxidoreductase I subunit M	0.0225
Cj0367c	*cmeA*	Multidrug efflux pump protein CmeA	4.7408	Cj1685c	*bioB*	Biotin synthase	0.0282
Cj0926		Membrane protein	4.1717	Cj1378	*selA*	L-seryl-tRNA(Sec) selenium transferase	0.0415
Cj0365c	*cmeC*	Multidrug efflux pump protein CmeC	3.9992	Cj0117	*pfs*	Aminodeoxyfutalosine nucleosidase	0.056
Cj1357c	*nrfA*	Cytochrome c nitrite reductase cytochrome c552 subunit	3.8977	Cj0314	*lysA*	Diaminopimelate decarboxylase	0.0587
Cj0318	*fliF*	Flagellar MS-ring protein	3.7812	Cj0002	*dnaN*	DNA polymerase III subunit beta	0.0627
Cj0946		Lipoprotein	3.6892	Cj1725		Periplasmic protein	0.0859
Cj0842		Lipoprotein	3.6419	Cj1171c	*ppi*	Peptidyl-prolyl cis-trans isomerase	0.0899
Cj0329c	*plsX*	Phosphate acyltransferase	3.4946	Cj1315c	*hisH*	Imidazole glycerol phosphate synthase subunit HisH	0.0975
Cj1215		Peptidase M23 family protein	3.4565	Cj1720		Hypothetical protein Cj1720	0.1062
Cj0277	*mreC*	Rod shape-determining protein MreC	3.3915	Cj0405	*aroE*	Shikimate 5-dehydrogenase	0.1123
Cj1573c	*nuoG*	NADH-quinone oxidoreductase subunit G	3.3741	Cj1041c		ATP/GTP-binding protein	0.1129
Cj0508	*pbpA*	Penicillin G-binding protein	3.2216	Cj1250	*purD*	Phosphoribosylamine–glycine ligase	0.1131
Cj0151c		Periplasmic protein	3.1399	Cj1670c	*cgpA*	Glycoprotein CpgA	0.1286
Cj1207c		Lipoprotein thiredoxin	3.1258	Cj1601	*hisA*	1-(5-phosphoribosyl)-5	0.1415
Cj0268c		Transmembrane protein	3.1233	Cj0440c		Transcriptional regulator	0.1449
Cj0734c	*hisJ*	Histidine-binding protein	3.0803	Cj0234c	*frr*	Ribosome recycling factor	0.1472
Cj0090		Lipoprotein	3.0552	Cj1516		Oxidoreductase	0.158
Cj0853c	*hemL*	Glutamate-1-semialdehyde aminotransferase	2.8635	Cj0014c		Integral membrane protein	0.159

*The proteome of these cells was compared to the proteome of a control culture not exposed to antibiotic. The proteins listed showed an abundance change of 2-fold or more and the q value was < 0.05*.

A parallel culture was not stained or analyzed by flow cytometry, and instead these cells were collected by centrifugation and re-suspended in fresh MHB and incubated for 24 h before staining with RSG and then analyzed using flow cytometry (Figure 2; +24 h MHB). In this antibiotic-free control we found a population of cells with a normally distributed range of fluorescence signal intensities from dim to bright. We found that the median signal intensity was reduced compared to the T0 culture.

In the test culture, we processed cells as detailed above but after centrifugation we re-suspended them in MHB containing 100 × MIC of penicillin G and then incubated for 24 h before staining with RSG. After flow cytometry analysis the penicillin G treated culture separated into three populations, labeled P1-P3 (Figure 2; +24 h penicillin G).

### Proteomic Analysis of *C. jejuni* With Different RSG Fluorescence Signals

We next analyzed the proteomic makeup of three biological replicates of cells incubated for 24 h in MHB (control) or three biological replicates of cells incubated for 24 h in MHB containing 100 × MIC of penicillin G. The control or penicillin-treated cultures were stained with RSG and sorted using FACS into cells that did not fluoresce, cells that fluoresced weakly or cells that fluoresced brightly (Figure 2). We collected broadly similar numbers of cells (1–3 × 10^6^) events in each of these groups and the collected cells were lysed and subjected to tryptic digests to release proteins and analyzed by mass spectrometry. Our results were also normalized according to the abundance of each protein relative to the total protein detected by mass spectrometry in each sample. The *C. jejuni* 11168H genome encodes 1,572 proteins and 95% (1,493) of these were detected in the combined untreated and penicillin G treated samples.

In the control cultures, we first compared the proteomes of the dimly stained cells and brightly stained cells. We did not identify differentially produced proteins, confirming that these were essentially the same population of cells. Considering that the dimly stained cells were the predominant population in the control culture, we next carried out a detailed comparison of the proteome of these cells with the proteome of the brightly stained cells in the penicillin G treated culture (Supplementary Table 1).

**Figure 3:**
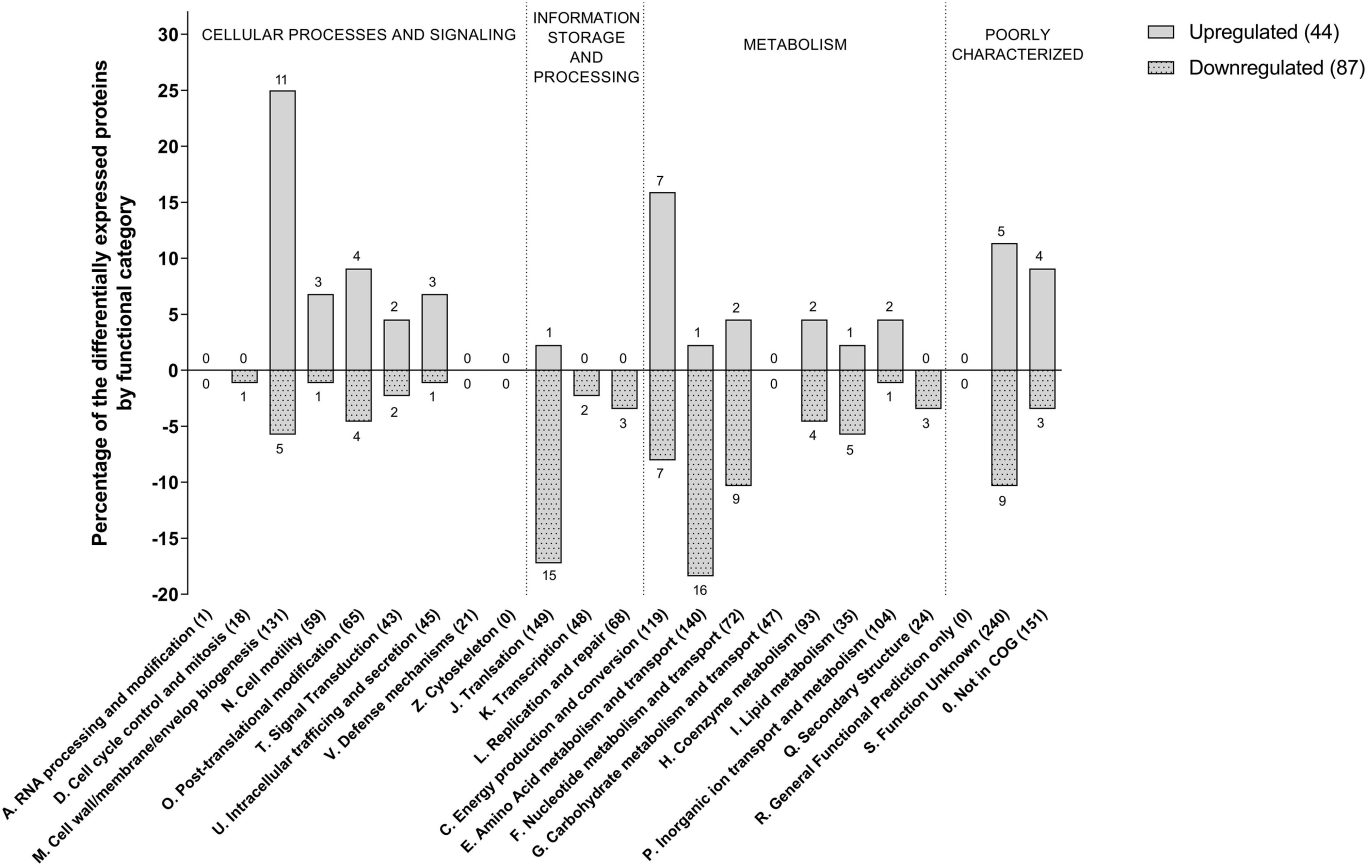
Functional COG categories of differentially produced proteins in penicillin G treated *C. jejuni* cells compared to untreated cells. Only significant produced proteins (q < 0.05) with abundance changes of 2-fold or more are shown.

We identified 1,331 proteins in the dimly stained cells from the control culture and 1,217 proteins in the brightly stained cells from the penicillin G treated culture. A comparison of these two datasets (Table 1) revealed 44 significantly more abundant and 87 significantly less abundant proteins in the brightly stained cells from the penicillin G treated culture. These proteins were assigned to COG functional categories (Figure 3). The cell membrane/envelope biogenesis and the energy production and conversion groups had the greatest number of proteins with increased abundance. These proteins included the CmeA and CmeC components of the CmeABC efflux pump, which has previously been associated with antibiotic, bile, heavy metals and other antimicrobials in *C. jejuni* (Iovine, 2013). The upregulation of this efflux pump has also been reported after phage infection (Sacher et al., 2018). Our proteome analysis also highlighted MurG (Cj1039) to be over-produced, which catalysis the final intracellular step of peptidoglycan synthesis (Shaku et al., 2020), indicating a possible role of this enzyme in restoring cell wall integrity after exposure to penicillin G. The majority of the proteins with increased abundance were associated with translation and amino acid metabolism and transport.

The increase in RSG fluorescence signal from bright cells after treatment with penicillin G indicates reduction of the dye within the cell. RSG stain will fluoresce when modified by bacterial reductases (Jaen et al., 2019) which are predominantly located in, or associated with, the electron transport chain of the inner membrane.

Analysis of the proteome of the penicillin G treated cells reveals that a component (NuoG, Cj1573c) of an unusual flavodoxin driven membrane-bound quinone-reductase Complex I (an NADH-quinone oxidoreductase but lacking the NADH dehydrogenase module subunits *nuoE* and *nuoF*) (Weerakoon and Olson, 2008) and a component (SdhA Cj0437) of a fumarate reductase (Cj0437-0439) (Taylor and Kelly, 2019) (previously mis-annotated as a succinate dehydrogenase) are significantly over-produced. These two key respiratory complexes are responsible for electron transfer to/from the menaquinone pool during respiration. The β (AtpD Cj1355) and δ (AtpH Cj0104) subunits of the F1 ATP synthase are also significantly over-produced.

In addition, the periplasmic facing cytochrome *c* nitrite reductase (NrfA, Cj 1357c) (Baymukhametov et al., 2018) was over-produced in penicillin treated cells. It is a periplasmic respiratory enzyme that couples to the formate dehydrogenase (Cj1508-1511), via menaquinol oxidation, in order to generate a membrane potential (Δp). NrfA is a pentaheme containing *c*-type cytochrome that catalysis the six-electron reduction of nitrite to ammonium by receiving electrons from menaquinol (Sellars et al., 2002). In *E. coli*, the NrfA nitrite reductase is normally produced during both anoxic and micro-oxic conditions, and in addition to reducing nitrite has also been shown to play a defensive role in NO detoxification (Poock et al., 2002). Our results show that NrfA in *C. jejuni* 11168H is significantly over-produced in penicillin G treated cells with a fold change of 3.9, and could provide a route to an alternative electron acceptor and protect against nitrosative stress (Pittman et al., 2007). The combined effects of these over-produced redox proteins undoubtedly contributes to the enhanced RSG intensity seen in the antibiotic treated cells and indicate a remodeling of the electron transfer chain toward a less electrogenic process. A potential shift from hydrogen or formate oxidation (which provides the highest measurable values of membrane potential of the available electron donors) to utilizing the flavodoxin driven membrane-bound quinone-reductase (Complex I) could help to dissipate the high membrane potential. Similarly, using periplasmic facing electroneutral terminal reductases such as NrfA or fumarate reductase, that accept electrons directly from the menaquinol pool rather than a QCR (Cj1184c−1186c), will also function to reduce the Δp as their H^+^/e^−^ = 0 (Taylor and Kelly, 2019). The net effect of these changes would modify the bioenergetics of the system and reduce the number of protons translocated per electron transfer, and thus aid to moderate Δp, ATP production and intracellular pH homeostasis. The reduction in the hyperpolarization of the cytoplasmic membrane could lead indirectly to restrain the ROS formation and reduce intracellular alkalization (Voskuil et al., 2018).

The efficacy of antibiotics being linked to changes in cellular respiration has been reported previously (Lobritz et al., 2015). In *E. coli* and *S. aureus* exposure to that treatment with bactericidal antibiotics gave rise to acceleration in respiration rate, and that inhibition of cellular respiration by creating a knockout mutant deficient in cytochrome *c* oxidase was sufficient to attenuate drug bactericidal activity. Furthermore, it was demonstrated that when the basal rate of electron transfer was accelerated, by uncoupling the electron transfer chain from ATP synthesis, the effectiveness of the antibiotic was increased. There is a general consensus that, for aerobes, the generation of reactive oxygen species (ROS) and the subsequent oxidative damage of many macromolecules is a known secondary effect of many bactericidal antibiotics including fluoroquinolones, beta-lactams and aminoglycosides (Dwyer et al., 2014).

The reason that potentiation of beta-lactam activity is less pronounced in bacteria grown at low oxygen levels may be because ROS levels are much lower under low oxygen aerobic conditions (Oh et al., 2015). Consistent with this suggestion, we (Hemsley et al., 2014) and others (Hamad et al., 2011) have shown that beta-lactam antibiotics are less effective in killing the Gram-negative bacterium *Burkolderia pseudomallei* under anaerobic compared to aerobic conditions. We would expect low levels of ROS in *C. jejuni* grown under microaerobic conditions. Also, the bacterium has a number of ROS detoxification enzymes (Taylor and Kelly, 2019) including; superoxide dismutase SodB (Cj0169), alkyl hydroxide reductase AhpC (Cj0334), catalase KatA (Cj1385), thiol peroxidases Tpx (Cj0779), bacterioferritin comigratory protein Bcp (Cj0271), two cytochrome *c* peroxidases (Cj0020c and Cj0358) and methionine sulfoxide reductases MsrA and MsrB (Cj0637 and Cj1112), none of which are significantly regulated upon treatment with penicillin G. Therefore, in summary we believe that unlike most bacteria studies to date, ROS do not play a major role in antibiotic killing in *C. jejuni* 11168H.

Persister cell formation in bacteria has previously been associated with reduced levels of metabolic activity and reduced membrane potential (Δp) and can be induced by perturbing the intracellular ATP levels. Although, recently it has been shown that the addition of salicylate can induce persister cell formation in *E. coli* via a mechanism that generates ROS (Wang et al., 2017). It has been suggested that the salicylate induces ROS generation and causes a decrease in membrane potential, which in turn leads to reduced metabolism and an increase in persistence. Further studies with *E. coli* using moderate levels of hydrogen peroxide (300–600 μM H_2_O_2_) as a direct source of ROS showed a protection against a lethal dose of ofloxacin by increasing persister cell formation by an order of magnitude (Vega et al., 2012). The metabolic burst we saw in penicillin G treated *C. jejuni* cells under microaerobic conditions could result from a remodeling of the electron transport chain to prevent hyperpolarization of the inner membrane and potentially curb intracellular alkalization and limit ROS formation. Therefore, the combination of these changes might play a broader role in supporting persister cell formation.

## Conclusion

In this study we set out to investigate how antibiotics affect *C. jejuni* 11168H and how this in turn might influence environmental survival. We report that *C. jejuni* 11168H forms persister cells after exposure to penicillin G. Another important finding from our study is the appearance of a population of cells with increased levels of redox proteins in cells exposed to penicillin G, resulting in a greater signal from cells stained with RSG. It is not clear if cells with increased levels of redox proteins are a feature of persister cells and further work would be required to explore this possibility.

## Data Availability Statement

The mass spectrometry proteomics data have been deposited to the ProteomeXchange Consortium via the PRIDE partner repository with the dataset identifier PXD021418.

## Author Contributions

The project was conceived by DS, OC, OS, and RWT. Experimental work was carried out by HM, OC, and RKT. Flow cytometry was enabled and supported by JL and RKT. Data analysis was carried out by AK-S, CB, HM, RKT, RWT, and ZY. Manuscript writing was carried out AK-S, CB, DS, HM, OS, RKT, RWT, SW, and ZY. All authors have read and approved this manuscript.

## Conflict of Interest

The authors declare that the research was conducted in the absence of any commercial or financial relationships that could be construed as a potential conflict of interest.
